# The use of electroencephalography in neurodegenerative disease and its utility in dementia

**DOI:** 10.1038/s44400-026-00089-5

**Published:** 2026-05-12

**Authors:** JiaoJiao Guo, Christos Panagiotis Lisgaras

**Affiliations:** 1 Department of Psychiatry, New York University Grossman School of Medicine, New York, NY USA; 2 The Nathan S. Kline Institute for Psychiatric Research, Center for Dementia Research, Laboratory of Brain Oscillations and Advanced Therapeutics, Orangeburg, NY USA

**Keywords:** Neurology, Neuroscience

## Abstract

Electroencephalography (EEG) offers a non-invasive window into brain function, often revealing abnormalities long before cognitive symptoms or neurodegeneration emerge. This review examines the expanding role of EEG in neurodegenerative disease and comorbid conditions that contribute to abnormal EEG. We review emerging EEG technologies that uncover previously underappreciated EEG abnormalities and outline future steps needed to advance EEG as a practical tool in clinical and research settings for dementia.

## Why EEG is important in neurodegenerative disease?

Dementia represents a rapidly advancing public health challenge^1^, fueled by the increasing prevalence of neurodegenerative diseases (ND), such as Alzheimer’s disease (AD), Parkinson’s disease (PD), dementia with Lewy bodies (DLB), and frontotemporal dementia (FTD)^2^. The genetic landscape contributing to these disorders is thought to involve many different genes^3^, but there are also other ND that are monogenic^4^ such as Huntington’s disease (HD), which is also characterized by progressive neurodegeneration^5^ and dementia^6^. Across these conditions, cognitive decline is common^6–9^ and is thought to emerge from progressive disruptions in brain circuits. In this context, changes in brain rhythms and large-scale network dynamics are increasingly appreciated as key contributors to the pathophysiology of ND and its cognitive sequalae^10^. Moreover, it has been suggested that different risk variants of AD can influence EEG power measurements^11^, suggesting that the heritability^12^ and/or de novo acquisition of different genetic variants influence brain rhythms recorded using electroencephalography (EEG)^13^.

EEG has long served as a non-invasive window into brain function and dysfunction^14,15^. Its high temporal resolution, broad accessibility, and relatively low-cost, position it as a valuable tool applicable to both clinical and research settings. Historically the EEG has often been used to access overt abnormalities such as background slowing, epileptiform activity and seizures^15^, particularly in the context of ND-comorbid conditions such as epilepsy, while offering limited yield in such EEG abnormalities in other psychiatric conditions^16^. However, advances in EEG technology, signal recording and processing, computational modeling, and machine learning (ML) have renewed interest in EEG as a relatively sensitive marker of early and subtle network deterioration across different ND^17^.

This review synthesizes current evidence on how EEG captures disrupted electrophysiological rhythms across major neurodegenerative dementias. We highlight the utility of EEG in differential diagnosis, its potential to identify prodromal or preclinical stages and the use of emerging biomarker candidates to improve prognostication and disease monitoring. Taken together, these perspectives highlight the rapidly expanding role of EEG in tracing neurodegenerative disorders at their roots with the potential to guide the development of early interventions^18^.

## Ways to record EEG activity

**Fig. 1:**
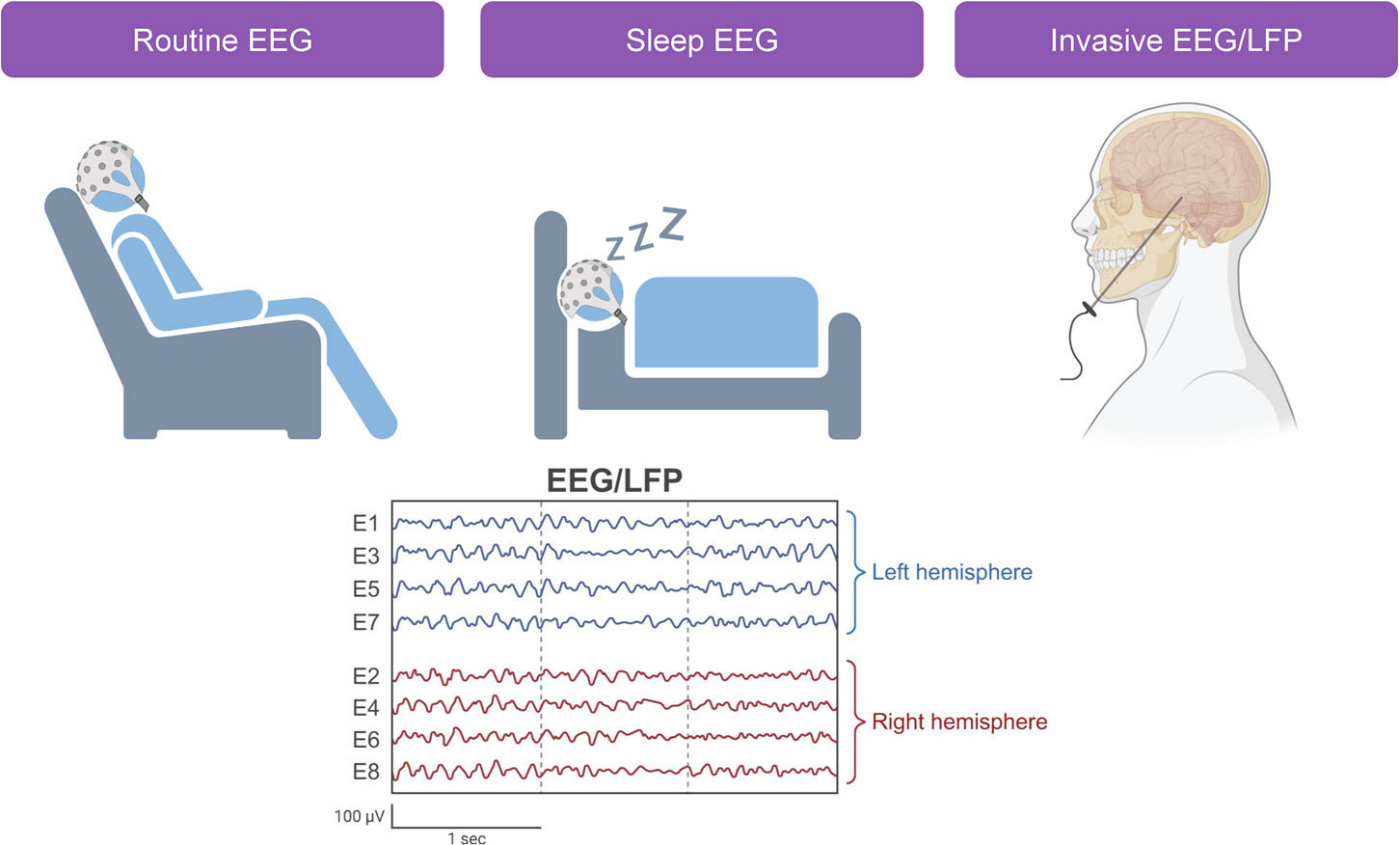
EEG recording modalities used in the context of neurodegenerative disease. Routine EEG is common and involves brief recordings, usually done in an outpatient setting. Sleep EEG (Polysomnography; PSG) is performed in dedicated rooms of a sleep laboratory and typically involve overnight recordings. Invasive EEG or local field potential recordings (LFPs) are typically rare but have been performed in the context of Alzheimer’s disease. These recordings used foramen ovale electrodes that aimed to reach deep cortical structures within the temporal lobe. Figure made using NYU Langone Health Biorender license.

EEG represents a neurophysiological technique that can be further classified by the recording conditions. In ND research, routine and sleep EEG are non-invasive scalp recordings widely used in human subjects (Fig. 1). In contrast, invasive EEG has so far been largely confined to animal models. However, there is a glaring exception where minimally invasive recordings^19^ uncovered silent seizures and other EEG abnormalities that were invisible on the scalp surface (Fig. 1)^20^. Here, we will primarily cover the use of EEG as a non-invasive tool to probe underlying circuit function and thus focus on scalp-recorded EEG modalities.

### Routine EEG

Routine EEG refers to resting-state and event-related recordings, distinguished by whether the use of stimuli is involved. Both these recordings are typically short in duration (~30 min to 1 h) and usually done in an outpatient setting. Clinically, EEG is often recorded alongside with electrooculography (EOG) and electrocardiography (ECG). Resting-state EEG captures spontaneous activity and provides indices of cortical arousal and vigilance in quiet wakefulness, which is informative in ND^21,22^. Event-related EEG complements this approach by measuring brain responses to a controlled stimulus. The readout of these analyses can include both continuous spectral power and event-locked waveforms. A key example is the P300, time-domain event-related potential (ERP) that is phase-locked to the stimulus and measured by estimating amplitude and latency^23^. The combination of resting-state and even-related recordings can improve the sensitivity of EEG markers and explain more behavioral variance than either modality alone.

### Sleep EEG

Sleep EEG is recorded as part of polysomnography (PSG), the standard method for assessing sleep in the clinic, where electromyography (EMG) is required besides EEG, EOG, and ECG. In this context, video recordings are synchronized with the PSG to aid review, especially for the identification of artifacts and clinically relevant events such as seizures. PSG recordings are typically performed in a sleep lab and last at least one full night (~8 h of recording). In sleep research, “sleep EEG” typically denotes the EEG component of PSG. Because sleep disturbances are a major risk factor for cognition deficits^24,25^, sleep EEG is highly useful for providing a window into the role of sleep in ND. More recently, wearable EEG sleep systems augmented by artificial intelligence (AI) have emerged as a novel approach for automated feature extraction and screening, as shown by Heremans et al.^26^. This approach could improve scalability and ecological validity while maintaining discriminative power, though the single EEG channel limitation and lack of external validation constrain its differential diagnostic utility.

### EEG combined with magnetoencephalography

EEG can be combined with magnetoencephalography (MEG), a modality that records the brain’s magnetic fields using sensors outside the head^27^. MEG is methodologically similar to EEG but it measures magnetic fields rather than voltages^28^. MEG is discussed here as it very often provides complementary or similar information to that obtained using EEG alone. However, it has also provided contrasting results, and we discuss below some instances that MEG localization may not be concordant to EEG-based localization, particularly in the case of aberrant network activity such as epileptiform activity. Nevertheless, compared to EEG, MEG is less affected by the conductivity of the skull and can significantly increase the spatial resolution of the recording, especially for oscillations of relatively low amplitude. However, it is a less cost-effective option, less portable and largely insensitive to radial and relatively deep structures. Notably, simultaneous EEG and MEG has been used in the context of epilepsy to better localize areas that seizures begin^29,30^, as well as in AD to determine network and oscillatory signatures underlying network hyperexcitability^31,32^, although such recordings were largely limited in capturing nocturnal sleep when such activities are typically most frequent.

## The spatial and temporal resolution of EEG

EEG can be recorded at multiple spatial and temporal scales, depending on electrode size, placement and sampling density (Table 1). Macroscale electrodes with (>1 mm contact diameter), as used in routine scalp EEG recordings provide broad brain coverage but relatively coarse spatial resolution. This limitation can be mitigated by increasing the number of electrodes, thereby enhancing overall spatial sampling. Mesoscale intracranial electrodes (50 µm to 1 mm), are less often used in the context of ND research; however, minimally invasive recordings with foramen ovale electrodes have been used in AD-dementia to uncover scalp-EEG silent seizures and epileptiform activity^20^.

**Table 1:** Comparison of traditional and emerging EEG technologies used in dementia of neurodegenerative disease

Category	Traditional EEG	Emerging EEG technologies
Spatial resolution	Low • 19–25 electrodes(10–20 system)	High • 64-256 electrodes(High-density arrays)
Sampling rate	Standard • 256–512 Hz	High to ultra-high • 1024–4000 Hz
Frequency band	Standard frequency bands • δ–γ (0.5–40 Hz) • Epileptiform activity	Wideband, includes • δ–γ (0.5–40 Hz) • Epileptiform activity • HFOs (>80 Hz)

*EEG* electroencephalography, *HFOs* high frequency oscillations.

The number of EEG electrodes can vary widely, however, in clinical practice, the 10–20 system with standard 19–25 scalp electrodes are the most commonly used. Alternative layouts such as the 10–10 and the 10–5 systems with different numbers of electrodes have also been utilized^33,34^. For even finer spatial resolution, high-density EEG and MEG systems with up to 256 electrodes/sensors are available for more detailed brain mapping^32,35^.

EEG signals are traditionally categorized into standard frequency bands: delta (**δ**) 0.1–4 Hz, theta (**θ**) 4–8 Hz, alpha (**α**) 8–12 Hz, beta (**β**) 12–30 Hz and gamma (**γ**) >30 Hz, collectively known as the “classic Berger frequencies.” Beyond these bands, high frequency oscillations (HFOs; 80–500 Hz) have been reported in individuals at risk of developing AD dementia^36^ as well as in sporadic AD cases^32^, expanding the range of abnormal brain rhythms implicated in AD-dementia. However, to accurately capture these signals and avoid aliasing, the EEG sampling rate must be at least 2–2.5 times the highest frequency of interest (i.e., for a brain rhythm of 500 Hz, sampling rate should be at least 1000 Hz). While routine clinical EEG is typically sampled at 256–512 Hz, wideband EEG targeting HFOs is still emerging, with some studies sampling up to 2048 Hz^36^, or even 4000 Hz^32^, enabling precise detection of these fast oscillatory events^37^.

In terms of signal analysis, visual EEG assessment and quantitative EEG (qEEG) methods are commonly used and will be discussed in the context of this review. Compared with visual EEG, qEEG reduces the subjectivity of visual interpretation and can capture features not readily apparent to the eye such as spectral power changes and oscillatory connectivity.

## Abnormal EEG in neurodegenerative disease—what does it tell us?

**Fig. 2:**
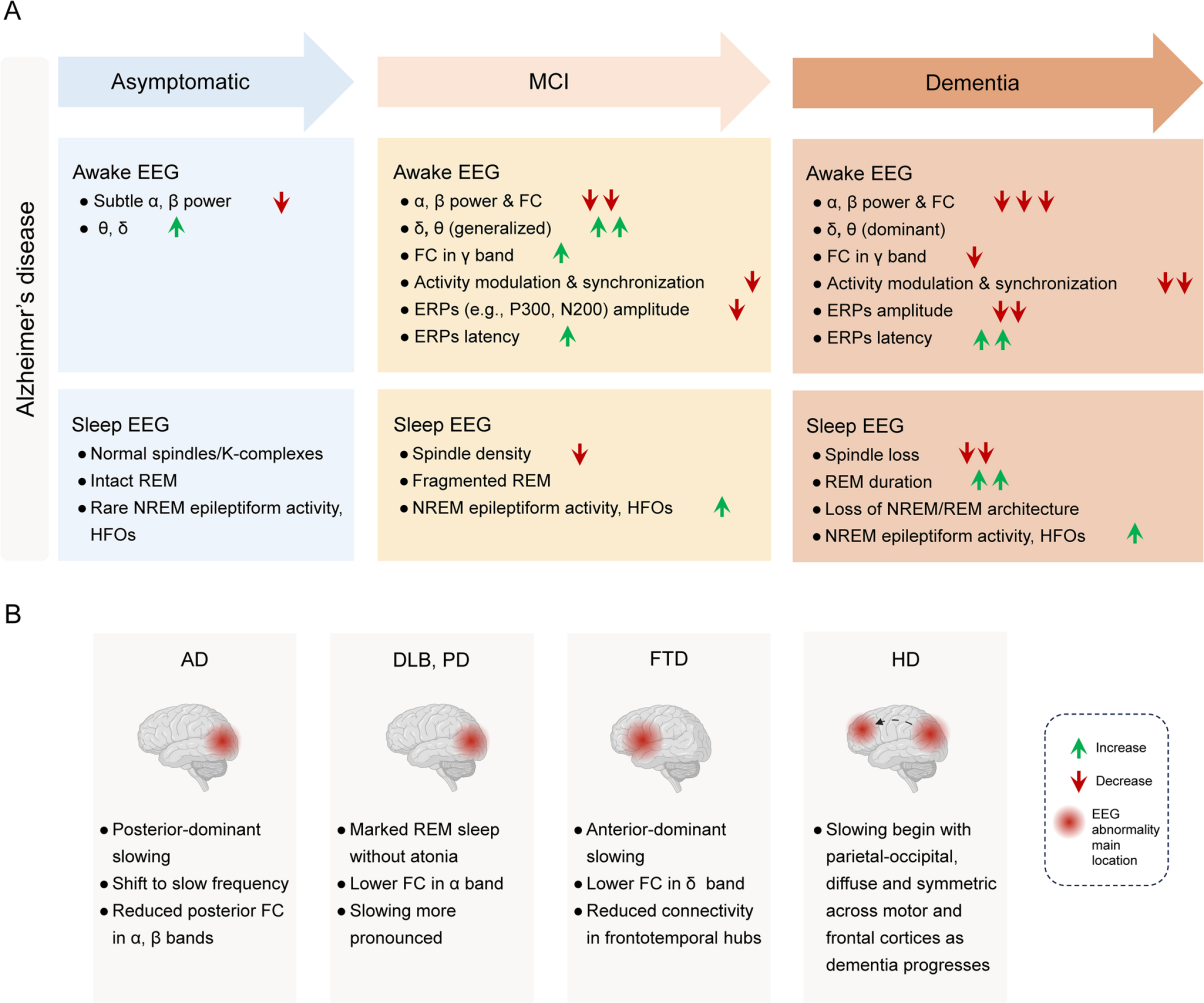
Spatiotemporal evolution and distinct profiles of EEG biomarkers in neurodegenerative disease. **A** Dementia development across clinical stages of Alzheimer’s disease (AD): Overview of EEG-related alterations spanning asymptomatic, MCI and dementia stages is shown. Awake EEG shows progressive background slowing (decreased **α** and **β** power, increased **δ,** **θ** dominance) and delayed ERPs. In addition, sleep EEG shows reduced spindle density, disintegration of NREM/REM sleep architecture, and often epileptiform activity (IEDs) and HFOs. Please note that underlying disease neuropathology may influence EEG-based trajectories, which are not covered in this overview. Also, such trajectories are mostly characterized in the context of AD and may differ across different etiologies. **B** Disease-specific differential characteristics: Relatively distinct EEG patterns reflecting regional characteristics are useful for differential diagnosis of AD dementia across different etiologies, including AD: posterior-dominant slowing. DLB & PD dementia: marked REM sleep without atonia, lower FC in **α** band and more severe slowing than AD. FTD: EEG slowing confined within anterior brain areas (e.g., frontal and temporal lobe), lower FC in **δ** band and reduced connectivity in frontotemporal hubs. HD: EEG slowing begins within parietal-occipital cortices, then becomes more diffuse and symmetric across motor and frontal cortices as HD progresses. AD Alzheimer’s disease, DLB dementia with Lewy bodies, ERP event-related potential, FC functional connectivity, FTD frontotemporal dementia, HD Huntington’s disease dementia, HFO high frequency oscillations, IEDs interictal epileptiform discharges, MCI mild cognitive impairment, NREM non-rapid eye movement sleep, PD Parkinson’s disease, REM rapid eye movement sleep. Figure made using NYU Langone Health Biorender license.

The EEG can provide a unique window into the spatiotemporal trajectory of cortical network dysfunction across the ND continuum. This is particularly valuable for identifying early signs of impending dementia at stages when cognitive or behavioral symptoms may still be subtle or absent. Importantly, it should be noted that EEG-derived biomarkers may not always follow the same temporal course as clinical symptoms or neuropathology. EEG abnormalities may precede, parallel, or lag behind cognitive decline, yet, at advanced dementia stages, they ultimately converge with synaptic failure and pronounced network dysfunction. These relatively progressive changes seen in EEG likely reflect loss of synaptic integrity, alterations in cortical excitability, and disruption of the coordinated oscillatory dynamics required for memory, attention and executive function. Thus, tracking these signatures across disease stages can offer mechanistic insight into brain network vulnerability and potential compensation. In the sections that follow, we review the evolution of EEG abnormalities during ND progression across four key domains: (i) resting-state rhythms, (ii) ERPs (iii) sleep EEG patterns, and (iv) functional connectivity (FC). The former (i.e., i, ii, iii) domains primarily describe local or regional EEG alterations, whereas FC focuses on network-level interactions between regions and is therefore discussed in a separate section. This evolution is also summarized in Fig. 2.

### Resting-state rhythms

Mounting evidence suggests that EEG may detect neurophysiological alterations even at the asymptomatic stages well before the onset of cognitive decline. In cognitively unimpaired normal individuals (*n* = 26) with PET-confirmed β-amyloid positivity (Aβ+), qEEG has shown decreased **α** and increased **δ** power^38^, indicating that EEG biomarkers may support early detection of asymptomatic AD, although further longitudinal studies are needed to confirm this finding. Similarly, Aβ+ cognitively normal individuals showed higher frontal **θ** and weak centroparietal **β** power, with more pronounced abnormalities in Aβ + mild cognitive impairement (MCI; ^39^). In cognitively normal individuals with PD, resting state eye-closed EEG revealed increased **θ** power and a reduced **α/θ** ratio compared with controls^40^, suggesting early electrophysiological slowing. In contrast, EEG biomarkers in asymptomatic DLB and FTD remain largely understudied^41^. For HD, findings appear inconsistent with reduced 7-8 Hz activity (~**θ/α**) power reported in asymptomatic carriers in one study^42^ but no such differences observed in another study, likely due to the different states of vigilance analyzed (eyes closed vs. not eyes closed)^43^.

Spectral slowing is considered one of the most robust and reproducible EEG hallmarks of dementia progression. Indeed, AD-related MCI and early stages of AD-dementia both show increased **θ** with reduced posterior **α** and lower **β** power^44^, suggestive of early thalamocortical dysregulation and posterior cortical involvement^45–47^. As AD-dementia develops, slowing becomes more generalized: **δ**-**θ** activity dominates, **α** power wanes, and the posterior rhythm collapses into a relatively low-frequency background^48,49^. However, it should be noted that EEG during the early stages of AD-dementia may not show changes in common frequency bands such as **δ** slowing^50^. In DLB- and PD-dementia similar but more pronounced slowing has been reported. Thus, posterior **α** peak diminishes or disappears, and the dominant rhythm is 1–2 Hz lower than what is seen in AD (~4–5 Hz) in both DLB^51,52^ and PD cases^53^. At the same time, between-dementia differences in mean spectral frequency or power have not been consistent, largely due to heterogenous study designs, EEG acquisition parameters and different processing regimes^52^. With increasing dementia severity, **δ**–**θ** power becomes even more prominent and the **α** peak often disappears^54,55^. In contrast, FTDs present a topographically distinct pattern-focal slowing confined to frontal and anterior temporal regions^56^, which topographically coincides with atrophy of these regions^57^. Notably, this slowing topography persists within frontal-temporal regions as FTD progresses, lacking generalization as in the case of AD-dementia^56^. This relatively distinct topography may assist in differentiating FTD- from AD-dementia, although overlap exists. For example, in a group of 24 patients with FTD, 42% preserved a normal posterior dominant rhythm, whereas 8% of patients showed complete absence of this normal rhythm, suggesting significant heterogeneity^58^. On the other hand, HD-dementia often begins with subtle narrow band slowing within the **δ** and **α** border (7–8 Hz) primarily over the parieto-occipital lobe, which later evolves into more diffuse and symmetric as motor and frontal cortices are affected^42,59,60^. Taken together, despite these common trajectories toward slowing, disease-specific topographies persist: posterior dominance in AD- and DLB-dementia, primarily frontal in FTD, and parieto-occipital in HD. Such preserved spatial signatures may assist in differential diagnosis, especially when combined with qEEG metrics of peak frequency and power ratio.

Biomarkers obtained from resting-state EEG have been investigated for their potential to predict the conversion from MCI to dementia, although it should be noted that some observations remain inconsistent. In a longitudinal study of 69 patients with AD-related MCI followed by approximately 14 months, **δ,** **θ,** and **α** coherence sources were stronger in those converted to AD vs. those that remained stable, suggesting that these measurements at a baseline could predict subsequent conversion to AD-dementia^61^. In contrast, Jelic et al.^62^ did not observe significant qEEG differences at baseline between future converters and non-converters when investigated the power of **α** and **θ** frequencies. However, some studies have reported reduced **α** power in AD-related MCI individuals who later converted to AD-dementia^63,64^. To improve predictive accuracy, Poil et al.^65^ combined multiple EEG biomarkers related to **β**-band activity into a diagnostic classification index. In their cohort of 86 patients initially diagnosed with AD-related MCI and followed for 2 years, 25 converted to AD-dementia^65^. Increased **β**-band activity was found to be predictive of conversion to AD-dementia^65^. Similarly, in a longitudinal study of PD, a subtype was identified at baseline that was characterized by elevated **δ** and **θ** power and reduced **α** and **β** power^66^. Over 5 years of follow-up, this subtype exhibited progressive increase in slow-frequency activity (i.e., **δ** and **θ** power) accompanied by cognitive decline, whereas motor-dominant subtypes remained relatively stable^66^. In a 3-year study of 47 patients with DLB-related MCI, posterior EEG slowing (<8 Hz) and increased dominant frequency variability (>1.5 Hz) were present in all 20 patients who converted to DLB, suggesting these abnormalities may predict progression to DLB^67^. Unlike the above ND types, resting-state EEG is not validated predictor of conversion in FTD and HD. However, qEEG measures, incorporating **β**/**θ** entropy metrics, may help distinguish FTD from controls or other dementias and could thus support prognostic stratification in longitudinal studies^68^.

### ERPs and induced oscillations

While spontaneous EEG activity offers a valuable means in assessing resting-state network function, event-related inductions of EEG oscillatory activity can provide complementary and, in some cases, more sensitive insights. One of the reasons is that some network disruptions may remain hidden until the “system” is actively perturbed. In this regard, ERPs are particularly informative, as they can sensitively index breakdown in large-scale oscillatory coordination during dementia development vs. spontaneous oscillations. In AD-dementia, N200 and P300 amplitude decreases and latency lengthens, coinciding with deficits in executive function and hippocampal atrophy^69–71^. Concurrent attenuation of task-evoked posterior **α** during working memory tasks was found for both AD-related MCI and AD cases vs. controls^72^, possibly reflecting weakened recruitment of associative networks. These flattened responses may reflect disturbances of long-range synchrony and inability to generate coherent oscillatory responses to external stimuli.

In DLB- and PD- dementia, delayed P300 responses and diminished mid-frontal **θ**/**δ** power ratio could indicate deficits in cognitive control and novelty detection, worsening with increasing dementia severity^23,73^. In FTD, P300 latency is delayed compared to AD-dementia, but not controls, suggesting it could be useful for differentiating FTD and AD-dementia cases^74^. Notably, this reduction in P300 latency has been correlated to worse cognition in FTD^75^. On the other hand, HD-dementia shows reduced event-related **θ** across many areas of the working memory network with **γ** responses inversely correlating with disease burden scores^76,77^.

ERPs and induced oscillations may also provide insights into the likelihood of conversion from AD-related MCI to AD-dementia. In a longitudinal study, 25 patients initially diagnosed with AD-related MCI and 11 age-matched controls were followed for 3 years^78^. Individuals with AD-related MCI who later converted to AD-dementia (*n* = 15) showed reduced **θ** activity during a word-processing task at baseline compared with MCI non-converters and controls^78^. A systematic review and meta-analysis including 4 longitudinal studies of AD-related MCI showed longer mean P300 latency and lower mean P300 amplitude between baseline and 1 year follow up measurements^79^, including cases that progressed (vs. those that remain cognitively stable)^80^. However, other studies have not found significant P300 differences between converters and non-converters. When such analyses combined β-amyloid levels the sensitivity and specificity were improved in discriminating those that converted to AD vs. those that remained MCI stable, underscoring the heterogeneity of ERP-based responses^81^. A meta-analysis of mainly cross-sectional studies found that reduced P300 amplitude and prolonged latency distinguishes cognitive stages in PD^82^, but their predictive value requires longitudinal validation. Similarly, a research gap exists in determining whether ERPs can predict cognitive conversion in DLB, FTD, and HD, given the absence of longitudinal validation.

### Sleep EEG patterns

Sleep EEG provides a sensitive measure of network integrity across ND, although specific alterations vary by underlying pathology. Along the AD continuum, reductions in non-rapid eye movement (non-REM) slow-wave activity and sleep spindle density have been reported in AD-related MCI, which correlated with β-amyloid and tau accumulation^83,84^. As dementia severity increases, both features further decline, possibly indicating impaired thalamocortical coupling and diminished synaptic homeostasis^85^. In DLB and PD, loss of REM atonia on PSG (i.e., REM sleep behavior disorder) serves as a characteristic early marker of underlying α–synuclein pathology and may precede dementia onset by years^86,87^. In PD, reduced slow wave activity during non-REM correlated with disease progression with high REM atonia predicting motor symptom progression^88^. FTD has been linked to pronounced sleep-wake and circadian disruption, and emerging data suggest altered spindle and sigma power (11–16 Hz) during non-REM sleep^89,90^. HD-dementia is characterized by reduced slow-wave sleep and increased nocturnal awakenings, along with fragmented sleep and unstable non-REM to REM cycling^91,92^.

By the dementia stage, sleep EEG converges across multiple etiologies of ND, although their magnitude, topography, and accompanying features remain largely disease specific. In AD-dementia, slow-wave (especially in N3-stage) and sleep spindles are profoundly reduced or absent^93^; **k**-complexes become rare, and REM is also reduced in duration^94^. DLB-dementia shows persistent REM without atonia, and frequent stage transitions^95^, alongside attenuated non-REM slow waves^96^. In FTD-dementia, sleep becomes highly fragmented with circadian disorganization and marked reductions in frontal sigma, spindles and slow-wave amplitude^97,98^. Patients with HD-dementia show shortened total sleep time, increased number of awakenings, and unclear EEG boundaries between the different sleep stages^99,100^.

Taken together, EEG abnormalities across dementias trace a consistent continuum: from focal inefficiency characterizing MCI to relatively diffused impairment in dementia. Slowing and reduced spindles at early disease stages may mark emerging thalamocortical dysfunction; subsequent loss of **α**–**β** rhythms, blunted ERPs, and flattened spectra could reflect large-scale disintegration of cortical assemblies. Despite differing molecular origins (β-amyloid-tau in AD, **α**-synuclein in DLB/PD, TDP–43 or tau in FTD, and mutant huntingtin in HD) the electrophysiological trajectory largely converges toward diminished temporal coordination and weakened long-range communication with important implications for memory and executive function.

### Sleep-predominant epileptiform activity

In contrast to the classical EEG abnormalities discussed above, epileptiform activity represents another EEG abnormality that primarily occurs during sleep. Sleep-predominant epileptiform activity is increasingly recognized as a contributor to ND, particularly AD-dementia^101^. Indeed, epileptiform activity, typically characterized by increased neuronal firing, has been shown to impact cognition in AD^102,103^, especially because it most commonly occur during sleep, a brain state when memory is typically consolidated^104^.

In early stages of AD, both interictal epileptiform discharges (IEDs) and seizures have been reported primarily during sleep^105^ and may contribute to fluctuations in cognitive function. Thus, identifying individuals with underlying epileptiform activity during sleep is important for their stratification in anti-seizure drug trials targeting cognitive outcomes^106^. However, it should be noted that not all patients with AD show epileptiform activity on routine or sleep scalp EEG as evidenced by foramen ovale recordings showing that scalp EEG recordings miss most of it^20^. Emerging markers of hyperexcitability, such as scalp HFOs, have been reported to occur in a broader population with AD-dementia^32^ and even in individuals at high risk of AD prior to the onset of cognitive decline^36^. Notably, such epileptiform patterns (IEDs, HFOs) are observed using either sleep EEG recordings or MEG^32,36^, but their spatial distribution may differ based on the modality. It has been shown that upon AD-dementia diagnosis, HFOs may be more widespread and thus may not show hemispheric lateralization, contrasting their right-predominant occurrence at early disease stages^36^. Notably, IEDs show an opposite hemispheric predominance, occurring primarily on the left hemisphere^36,102,107^, a result that appeared to contrast MEG findings that showed that IEDs predominantly occur on the right hemisphere^32^. Although the exact reason why EEG and MEG may show different hemispheric lateralization is unclear, both approaches, especially when conducted simultaneously, may yield complementary information than either approach conducted alone.

It should also be noted that IEDs have also been reported to occur in other ND, besides AD-dementia, including DLB^108^ and FTD^109^. Overall, these findings highlight that sleep-related epileptiform activities could be valuable biomarkers across different ND and could aid in earlier diagnosis and patient stratification to anti-seizure drug trials.

### Functional connectivity

ND are characterized by profound functional disconnection between connected brain regions. EEG-based connectivity analysis models the relationship between neural activity across different regions, revealing changes in temporal dynamics between interacting brain regions. Importantly, altered EEG-measured FC has been found linked to the progression of ND, suggesting that it could serve as a biomarker^110^. This significant clinical potential has led to the development of over thirty tools of neurophysiological connectivity analysis^111^.

In a comparative study of EEG-based features across 434 patients with AD/PD and 499 controls, researchers found that FC deviations occurred in approximately 80% of patients with AD/PD^112^. In the context of AD, decreased EEG-based FC is commonly reported in **α** frequency band^113^. On the other hand, findings in **θ** frequency are mixed^114^, likely explained by regional heterogeneity between AD and FTD^115^. Lastly, evidence regarding **γ**-band FC is also heterogeneous. Increased FC in **γ** has been reported in AD-related MCI but decreased in AD-dementia, suggesting that increased **γ** activity could serve as a potential marker for prodromal AD^114^. However, reduced **γ** band FC can also be observed in AD-MCI depending on subgroup characteristics^116^, and increased event-related **γ** FC has also been reported in AD-dementia^117^.

FC patterns in DLB- and PD-dementia show both similarities and differences compared to AD-dementia. In DLB-dementia, **α** connectivity is typically reduced, but often more severely affected than in AD-dementia^118^, albeit some studies reported no **α** difference between DLB-related MCI and AD-related MCI^119^. DLB-dementia further distinguishes itself from AD-dementia by exhibiting weaker posterior-anterior **β** connectivity and greater **θ**-band network segregation although this pattern appears method-dependent^120^. Conversely, despite sharing some underlying pathology with DLB, PD-dementia is characterized by relatively increased FC in **β** frequency band^121,122^, although **β**-band findings remain heterogenous, especially across brain regions^123,124^. Evidence for **γ**-band FC in PD is limited, but increased **γ** coherence has been noted in PD-related MCI^125^. One study uncovered a sex-related FC difference in PD-related MCI: men, but not women, showed reduced FC within the **α** frequency band^126^.

Compared with AD-dementia, FTD has been associated with lower **δ**-band^127^, in line with findings from an automatic stacked-model classification approach^128^. In contrast, **α**-band FC did not reliably discriminate FTD from AD-dementia in the same model, although reduced **α** FC was found^128^. Using a multidimensional FC framework, reduced connectivity across mid- and long-range frontotemporal hubs was reported in FTD but not in AD-dementia^129^, suggesting disease-specific disruption of frontotemporal networks. In comparison, EEG based FC studies in HD-dementia remain scarce compared to other ND according to a systematic review^130^. Existing evidence suggests global network disruption, with reduced overall FC reported in HD-dementia^131^, alongside increased **δ**-band FC relative to controls^132^. However, the limited number of studies challenges the reliable identification of a consistent FC signature in HD-dementia.

The choice of FC measure can influence the findings of EEG-based studies in ND. Specifically, inconsistent results have been reported due to the use of different FC measures with varying sensitivity to volume conduction. For instance, Stam et al.^133^ reported reduced **β** band phase lag index (PLI) and imaginary coherence in patients with AD-dementia (*n* = 15) compared with cognitively normal controls (*n* = 13), while no significant difference was found using phase coherence when data were analyzed separately for short and long distance electrode pairs. In a larger cohort, Briels et al.^134^ investigated the reproducibility of six FC measures in 809 patients with probable AD and subjective cognitive decline and found that leakage-corrected amplitude envelope correlation showed higher robustness in **α** and **β** band, whereas PLI and weighted-PLI did not consistently show reductions in these frequency bands. Overall, these observations suggest that conflicting findings in FC may partially stem from different FC approaches and/or specific frequency bands analyzed.

Overall, the data indicates that FC is reduced across ND. However, FC findings are highly variable, depending on pathology subtypes, frequency bands, disease stage, specific brain area, and other factors (e.g., sex, age). Given this heterogeneity, advanced techniques like ML and deep learning would be valuable in automatically extracting complex features^135^. Notably, by integrating multiple features, these models have shown promising performance in dementia subtype differentiation in selected datasets, with classification accuracy exceeding 90%^136^, although such results require validation in larger cohorts.

## Concluding remarks

To conclude, the EEG provides a non-invasive and practical tool to detect and longitudinally monitor network brain dysfunction across different ND. Its cost-effectiveness and scalability are advantageous across different settings that may lack state-of-the-art equipment. Several metrics could be extracted using simple methods such as changes in spectral power and FC that can be complemented by more advanced analytical methods to ascertain how these progressive disorders alter cortical communication. This multifaceted utility of the EEG could offer benefits across several clinical domains.

First and foremost, EEG can serve as a potential tool for early disease detection. Given that the pathophysiological processes of AD can silently begin up to 20 years before symptoms arise^137^, early diagnosis is crucial for designing interventions aiming at slowing disease progression as early as possible^138^. In this context, EEG combined with advanced signal analysis approaches have shown promising ability to differentiate MCI from healthy controls, highlighting its potential as a non-invasive biomarker for early cognitive decline^139,140^, although validation in larger cohorts will be beneficial. Complementary methods such as electrical source imaging reconstruction of cortical generators from scalp EEG may enhance the anatomical interpretability of EEG alterations^141^. Furthermore, as AD develops, an overall decrease in the power of oscillations and FC represents a progressive change that can be reliably tracked by longitudinal EEG recordings.

EEG provides useful markers for differentiating dementias across different ND. Because approximate 70% of dementia is due to AD, most EEG studies focus on distinguishing AD from other etiologies^111^. A striking contrast is dominant-rhythm slowing: frequencies <8 Hz occur in 90% of DLB but only 10% of patients with AD^52^. Importantly, sleep can aid dissociating different AD subtypes, which may be linked to different vulnerabilities in subcortical nuclei controlling sleep and wakefulness across subtypes^142^.

An additional area that EEG is helpful is prognosis and treatment monitoring. Prospective studies show that EEG can predict evolution from AD- and PD-related MCI to dementia^143^. In PD, **δ**-band power increases from cognitively normal PD to MCI to PD-dementia, paralleling disease progression^144^. For therapy monitoring, P300 latency is sensitive to drug effects in cognitive performance^23^. In this context, increased P300 amplitude and shorter latency parallel better cognitive scores in drug-treated patients with AD^145,146^.

However, several caveats should be acknowledged when using EEG for diagnosis, especially in the asymptomatic or early disease stages. First, EEG patterns also change with normal aging^147^, especially after age 60. Therefore, physiologically aberrant rhythms due to aging must be carefully dissociated from early pathological signs of a disease-related context. Second, EEG abnormalities are neither present within a single frequency band nor exclusive to a single ND type. Indeed, shared patterns may represent common pathophysiological mechanisms underlying cognitive decline among different ND, while differences may reflect disease-specific variability. Third, other factors including age, sex and environmental factors could influence neural plasticity and in turn EEG features that could confound EEG biomarker interpretation. Integrating EEG with advanced analytic methods (e.g., ML) and multimodal imaging (e.g., magnetic resonance imaging; MRI and/or diffusion tensor imaging; DTI, focused on structural connectivity) could improve early detection and differential diagnosis of ND.

## Limitations

Lastly, we would like to acknowledge a couple of limitations. First, given the broad scope of EEG biomarkers across ND, the details for each condition are limited and may emphasize more extensively studies related to AD-dementia. Second, as a narrative review, no formal sample size threshold was applied, which may limit the number of studies reported here despite prioritizing robust studies such as systematic reviews and meta-analyses. Third, we focused primarily on EEG features, whereas emerging computational methods such as graph-theoretical and source-based analyses were not discussed in depth, but we consider them important for future implementation. Future multicenter studies using standardized EEG protocols and advanced analytical frameworks could help establish more reliable EEG-based and multimodal biomarkers for early diagnosis and discrimination of disease stages.

## Data Availability

No datasets were generated or analyzed during the current study as this is a review article.
